# Identification and Validation of the Prognostic Stemness Biomarkers in Bladder Cancer Bone Metastasis

**DOI:** 10.3389/fonc.2021.641184

**Published:** 2021-03-19

**Authors:** Yao Kang, Xiaojun Zhu, Xijun Wang, Shiyao Liao, Mengran Jin, Li Zhang, Xiangyang Wu, Tingxiao Zhao, Jun Zhang, Jun Lv, Danjie Zhu

**Affiliations:** ^1^ Department of Orthopedics, Zhejiang Provincial People’s Hospital, Hangzhou, China; ^2^ Department of Orthopedics, Hangzhou Medical College People’s Hospital, Hangzhou, China; ^3^ Department of Musculoskeletal Oncology, Sun Yat-Sen University Cancer Center, Guangzhou, China; ^4^ State Key Laboratory of Oncology in South China, Guangzhou, China; ^5^ Collaborative Innovation Center for Cancer Medicine, Guangzhou, China; ^6^ Department of Head and Neck Surgery, Sun Yat-Sen University Cancer Center, Guangzhou, China

**Keywords:** bladder urothelial carcinoma, bone metastasis, cancer stem cell, mRNAsi, prediction model

## Abstract

**Background:**

Bladder urothelial carcinoma (BLCA) is one of the most common urinary system malignancies with a high metastasis rate. Cancer stem cells (CSCs) play an important role in the occurrence and progression of BLCA, however, its roles in bone metastasis and the prognostic stemness biomarkers have not been identified in BLCA.

**Method:**

In order to identify the roles of CSC in the tumorigenesis, bone metastasis and prognosis of BLCA, the RNA sequencing data of patients with BLCA were retrieved from The Cancer Genome Atlas (TCGA) databases. The mRNA expression-based stemness index (mRNAsi) and the differential expressed genes (DEGs) were evaluated and identified. The associations between mRNAsi and the tumorigenesis, bone metastasis, clinical stage and overall survival (OS) were also established. The key prognostic stemness-related genes (PSRGs) were screened by Lasso regression, and based on them, the predict model was constructed. Its accuracy was tested by the area under the curve (AUC) of the receiver operator characteristic (ROC) curve and the risk score. Additionally, in order to explore the key regulatory network, the relationship among differentially expressing TFs, PSRGs, and absolute quantification of 50 hallmarks of cancer were also identified by Pearson correlation analysis. To verify the identified key TFs and PSRGs, their expression levels were identified by our clinical samples *via* immunohistochemistry (IHC).

**Results:**

A total of 8,647 DEGs were identified between 411 primary BLCAs and 19 normal solid tissue samples. According to the clinical stage, mRNAsi and bone metastasis of BLCA, 2,383 stage-related DEGs, 3,680 stemness-related DEGs and 716 bone metastasis-associated DEGs were uncovered, respectively. Additionally, compared with normal tissue, mRNAsi was significantly upregulated in the primary BLCA and also associated with the prognosis (P = 0.016), bone metastasis (P < 0.001) and AJCC clinical stage (P < 0.001) of BLCA patients. A total of 20 PSRGs were further screened by Lasso regression, and based on them, we constructed the predict model with a relatively high accuracy (AUC: 0.699). Moreover, we found two key TFs (EPO, ARID3A), four key PRSGs (CACNA1E, LINC01356, CGA and SSX3) and five key hallmarks of cancer gene sets (DNA repair, myc targets, E2F targets, mTORC1 signaling and unfolded protein response) in the regulatory network. The tissue microarray of BLCA and BLCA bone metastasis also revealed high expression of the key TFs (EPO, ARID3A) and PRSGs (SSX3) in BLCA.

**Conclusion:**

Our study identifies mRNAsi as a reliable index in predicting the tumorigenesis, bone metastasis and prognosis of patients with BLCA and provides a well-applied model for predicting the OS for patients with BLCA based on 20 PSRGs. Besides, we also identified the regulatory network between key PSRGs and cancer gene sets in mediating the BLCA bone metastasis.

## Introduction

Bladder urothelial carcinoma (BLCA) is the most common urinary system malignancies, with a high mortality and male predominance (1). With regard to localized disease, surgical treatment, or radiotherapy can be used with a favorable prognosis (2). However, as for metastatic BLCA, these therapeutic options often achieve limited effects in controlling the disease progression (3). Bone is a common metastatic site of BLCA, and the osteolytic destruction often induces skeletal-related events (SREs), such as local pain, pathologic fracture and even spinal cord compression (4). Thus, distant metastasis, in especial bone metastasis, has become the main cause which decreases the overall survival (OS) of patients with BLCA. Facing this clinical dilemma, it is important to investigate the potential tumorigenic and metastatic mechanism of BLCA, and subsequently identify its prognostic biomarkers and therapeutic targets.

The cancer cell population includes various tumor cells, cancer stem cells (CSCs) and microenvironment cells which make the heterogeneity (5). CSCs are specific cell types of malignancies and exhibit stem‐like properties, such as self-renewal and initiating other types of cells (6). They are regarded as the main drivers of tumorigenicity, metastatic dissemination and treatment resistance (7). In BLCA, the relationship between molecular biology and CSCs has been addressed, and CSCs are regarded as an important factor inducing tumor recurrence and chemotherapeutic agents resistance (8). However, their roles in the distant metastasis of BLCA, especially bone metastasis, are still unclear.

With the development of bioinformatics, the features of CSC can be identified by deep learning methods which assist scientists in evaluating oncogenic dedifferentiation (9). Two indices have been proposed, namely mRNA expression-based stemness index (mRNAsi) and DNA methylation-based stemness index (mDNAsi). The former reflects the stemness gene expression and the latter shows the stemness epigenetic characteristics. Both of them have been proved to be associated with CSCs activity, along with tumor dedifferentiation and pathological grade (10). However, its roles in BLCA have not been identified, neither is bone metastasis.

In this study, RNA-seq data and clinical information of BLCA samples were collected from The Cancer Genome Atlas (TCGA) databases and the differential expressed genes (DEGs) were identified, along with the association between mRNAsi and tumorigenesis, bone metastasis and patients’ OS. The prognostic stemness-related genes (PSRGs) were also found and based on them, we constructed the predict model. Moreover, the regulatory mechanism of PSRGs and downstream signaling pathway were also explored to provide the prognostic biomarkers and therapeutic targets which may assist oncologists in the prediction of BLCA occurrence and bone metastasis, along with the clinical treatment.

## Method

### Data Extraction

In formats of raw-counts and Fragments Per Kilobase per Million (FPKM), RNA sequencing data of 411 primary BLCA samples and 19 normal solid tissue samples were downloaded from The Cancer Genome Atlas (TCGA) (https://tcga-data.nci.nih.gov). Bone metastasis diagnosis was specifically concerned, and other selected potential factors include demographics data (i.e., age at diagnosis, race, and gender), tumor information (i.e., histologic grade, AJCC clinical stage, TNM classification), and endpoint data (i.e., OS status and OS time). All of them were extracted in eXtensible Markup Language (XML) files from the database.

### The Estimation of mRNAsi

In the current study, the normalized gene expression profiles of each sample were used to estimate the mRNAsi by the algorithm named one-class logistic regression machine learning (OCLR). In the original article of mRNAsi, Malta, T.M., et al. reported mRNAsi as an index between 0 and 1, which could evaluate the activity of CSCs, dedifferentiation of malignant cells (9).

### Differential Expression Analysis and Functional Enrichment Analysis

Statistical analysis began with four different groups of DEGs analysis by edgeR algorithm. |log2 Fold Change (FC)| > 1.0 and False Discovery Rate (FDR) value < 0.05 were used as the screening criteria in the identification of DEGs. The groups of DEGs were as follows: primary BLCA vs normal solid tissue; Stage I/II BLCA vs Stage III/IV BLCA; low mRNAsi BLCA vs high mRNAsi BLCAs (divided by the median mRNAsi); BLCA without bone metastasis vs BLCA with bone metastasis.

The Gene Ontology (GO), Kyoto Encyclopedia of Genes and Genomes (KEGG) enrichment analysis as well as Gene Set Enrichment Analysis (GSEA) were used to explore the potential signaling pathways in the tumorigenesis, progression and metastasis (11). Both of them were conducted in our study. GO analysis can illuminate the biological processes (BPs), cellular components (CCs), and molecular functions (MFs) of enriched DEGs, and KEGG analysis describes the pathophysiologic pathways while 50 hallmarks of cancer gene sets were retrieved from Molecular Signatures Database (MSigDB) v7.0 (https://www.gsea-msigdb.org/gsea/msigdb/index.jsp) for GSEA (12).

### The Identification of PRSGs

The intersection of identified DEGs in these four groups were integrated into the univariate Cox regression analysis. Genes with p<0.05 in the univariate Cox regression analysis were defined as PRSGs. Next, all the PRSGs were included in the multivariate Cox analysis. The Least Absolute Shrinkage and Selection Operator (LASSO) regression analysis were utilized to ensure no overfitting of the final multivariate Cox model and the five-fold cross-validation was performed. In terms of model diagnosis, the discrimination and goodness of fit (GOF) of the multivariate model were illustrated by the area under curve (AUC) of receiver operator characteristic (ROC) curve for five-year OS and the Cox-Snell residual plot, respectively.

### The Calculation of Prognostic Index (PI) and Independent Prognosis Analysis

The formula of the multivariate Cox model (as followed) was used to calculate the PI for each BLCA patient.

$$\text{P}{\text{I}}_{m}=\beta 1\times \text{PRS}{\text{G}}_{1}+\beta 2\times \text{PRS}{\text{G}}_{2}+\beta 3\times \text{PRS}{\text{G}}_{3}\ldots \;\ldots +\beta \text{n}\times \text{PRS}{\text{G}}_{n}$$

In the formula, “m” represented the number of each BLCA patient; “n” represented the number of prognostic PRSG in the multivariate model; “β” represented the coefficient of each PRSG in the multivariate model. In addition, all BLCA patients were divided into the high-risk group or low-risk group according to the median of PI. Moreover, the independent prognosis value of the PI in BLCA was evaluated by Kaplan-Meier survival analysis, univariate Cox analysis and multivariate Cox analysis corrected by age at diagnosis, gender and AJCC clinical stage.

### The Construction of the Prognostic Nomogram

The Cox models including PI were used to construct the prognostic nomogram, which could predict the 3-, 5- and 8-year OS probability of BLCA patients. The calibration plot was used to illuminate the calibration of the prognostic nomogram. The decision curve and time-related ROC with 95% confidence interval were also conducted to illustrate the patient benefit and the discrimination of the nomogram.

### The Identification of Transcription Factors (TFs) and Signaling Pathways Co-Expressed With PRSGs

First of all, official gene symbol of 318 cancer related TFs, 50 hallmarks of cancer gene sets were retrieved from Cistrome database (http://cistrome.org/) and Molecular Signatures Database (MSigDB) v7.0 (https://www.gsea-msigdb.org/gsea/msigdb/index.jsp), respectively (12, 13). Absolute quantification of the 50 hallmark of cancer gene sets in all the samples were quantified as continuous variables by Gene Set Variation Analysis (GSVA) (14). Then, the co-expression analysis was performed among differential expressed TFs, PRSGs and absolute quantification of 50 hallmarks of cancer. Interaction pairs between TFs and PRSGs with |correlation coefficient| > 0.40 and P value < 0.05 along with interaction pairs between PRSGs and hallmarks of cancer with |correlation coefficient| > 0.25 and P value < 0.05 were used to construct the regulatory network among TFs, PRSGs, and hallmarks of cancer.

### Immunohistochemistry (IHC) Validation

The IHC slides and information were obtained from the Human Protein Atlas. Immunostaining on each slide was assessed by experienced pathologists to examine the percentage of EPO, ARID3A, CGA, and SSX3 positive tumor cells (11).

### ATAC-Seq

Assay for Targeting Accessible-Chromatin with high-throughout sequencing (ATAC-seq) data available from TCGA GDC (https://gdc.cancer.gov/about-data/publications/ATACseq-AWG) were used to validated the regulation mechanism of key PRSGs (15). Gviz package of Bio-conductor were used to visualize the accessible peaks (16).

### Statistics Analysis

Discontinuous variables should be presented as percentages while continuous variables in normal distribution should be described as mean ± standard deviation (SD) or else reported as median (Range). Variance homogeneous and normal distributed continuous variables could be compared by student t-test, otherwise, the Mann-Whitney U-test or Kruskal-Wallis H-test should be used. In this study, only two-sided P value < 0.05 was considered as statistically significant for all analysis process. The R software (www.r-project.org; version 3.6.1; Institute for Statistics and Mathematics, Vienna, Austria) were used for all statistics analysis processes.

## Results

### DEGs Analysis and Functional Enrichment Analysis

**Figure 1:**
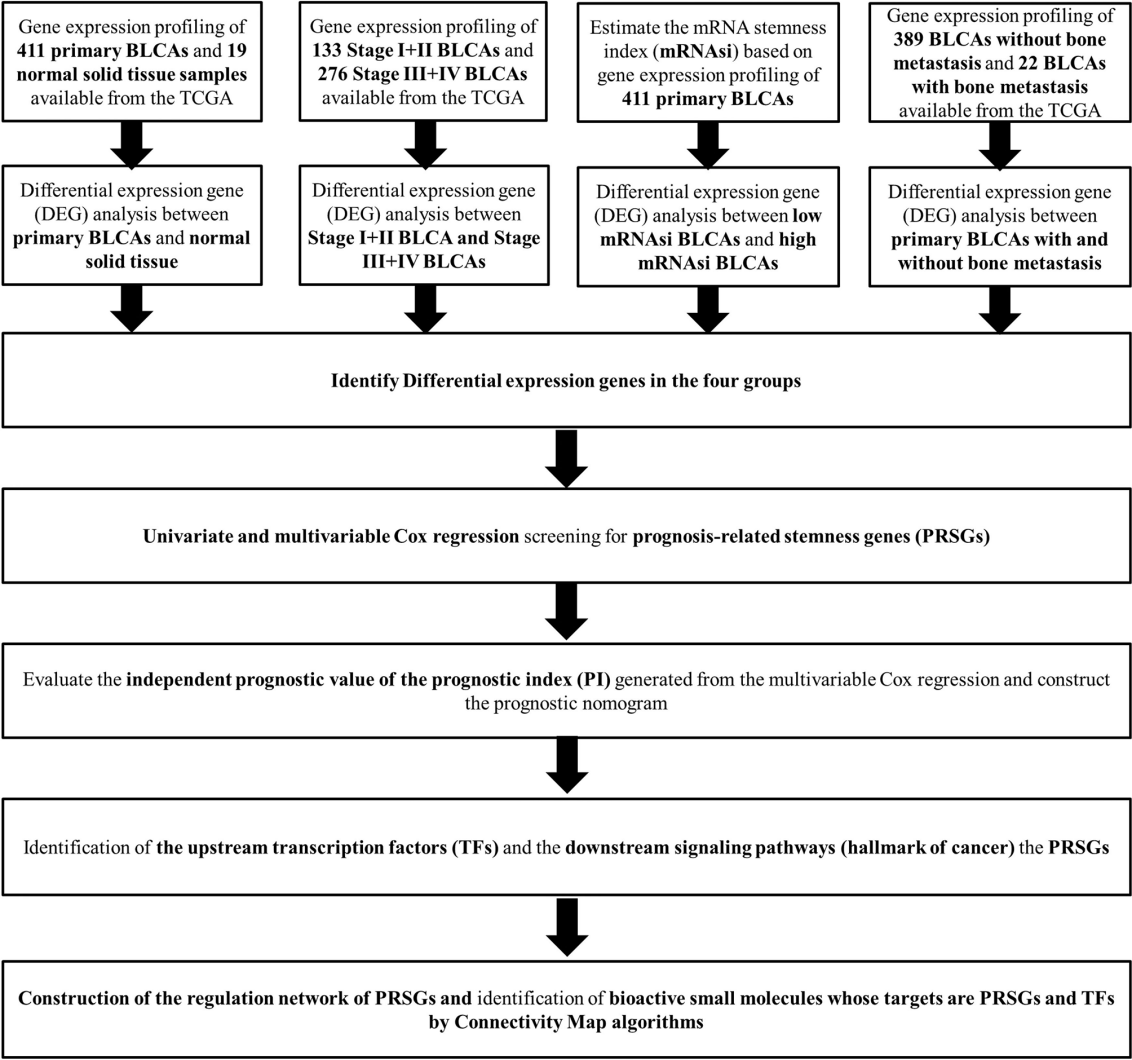
The flowchart of all analysis processes.

**Figure 2:**
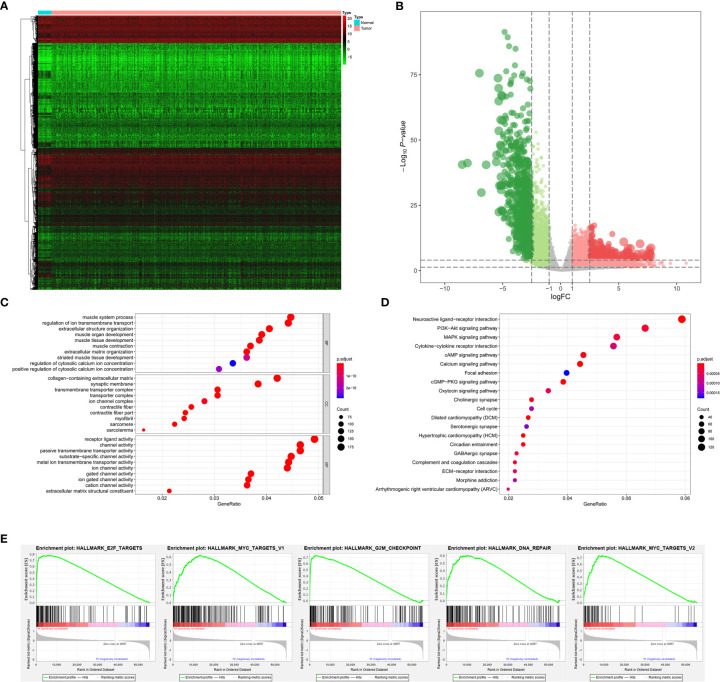
The results of differential expression genes analysis and functional enrichment analysis between primary BLCAs and normal solid tissue samples: The heatmap **(A)** and volcano plot **(B)** of the differential expressed genes. The GO **(C)**, KEGG **(D)**, and **(E)** top five GSEA enriched terms for the differential expressed genes.

The analysis process of the current study was summarized in the flowchart (**Figure 1**). In DEG analysis, all RNA-seq data were from primary BLCA samples or normal solid tissues, not distant metastasis tumors. All samples with missing grouping information (normal or tumor; stage I/II or stage III/IV; low mRNAsi or high mRNAsi; primary BLCAs without bone metastasis or primary BLCAs with bone metastasis) were deleted. A total of 8,647 genes (2,949 downregulated genes and 5,698 upregulated genes) were identified as DEGs between 411 primary BLCAs and 19 normal solid tissue samples in the heatmap (**Figure 2A**). The volcano plot of these DEGs were presented in **Figure 2B**. In order to explore the features of identified DEGs, GO, and KEGG analysis were used. The significant enrichment items of biological processes (BPs), cellular components (CCs), molecular functions (MFs) were muscle system process, collagen-containing extracellular matrix, and receptor ligand activity, respectively (**Figure 2C**). The KEGG pathways identified neuroactive ligand-receptor interaction, PI3K-Akt signaling pathway, MAPK signaling pathway. and cytokine-cytokine receptor interaction as the key enriched signaling pathways (**Figure 2D**). And the enrichment plots of top five significant Hallmark gene set in GSEA were shown in **Figure 2E**, illustrating that DNA repair, myc targets, E2F targets and G2M checkpoint were the most significant enrichment items.

**Figure 3:**
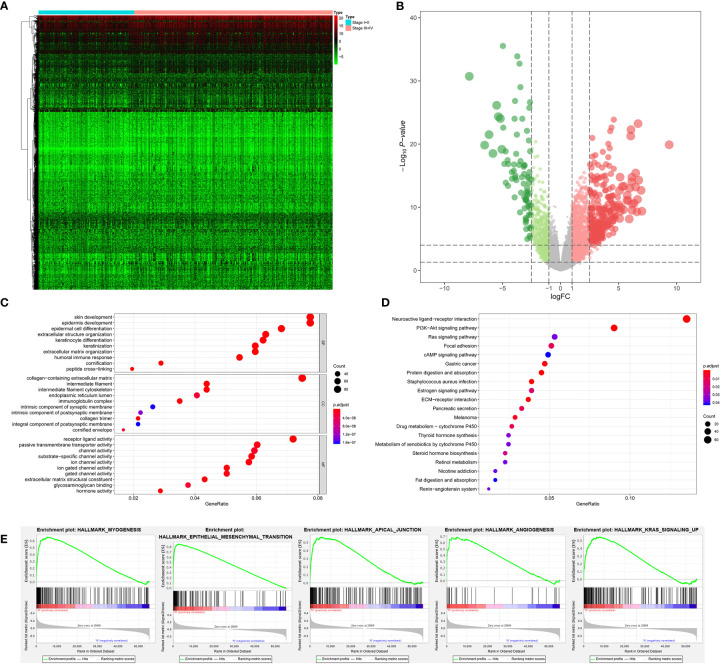
The results of differential expression genes analysis and functional enrichment analysis between Stage I or II BLCAs and Stage III or IV BLCAs: The heatmap **(A)** and volcano plot **(B)** of the differential expressed genes. The GO **(C)**, KEGG **(D)**, and **(E)** top five GSEA enriched terms for the differential expressed genes.

Among 389 primary BLCA samples, 113 Stage I/II BLCAs were identified, along with 276 Stage III/IV BLCAs. A total of 2,383 DEGs (700 downregulated ones and 1,683 upregulated ones) were uncovered between Stage I/II and III/IV BLCAs. The heatmap and volcano plot were shown in **Figures 3A, B**. The GO enrichment items included skin development, collagen-containing extracellular matrix, receptor ligand activity (**Figure 3C**). KEGG analysis uncovered the roles of neuroactive ligand-receptor interaction, PI3K-Akt signaling pathway and Ras signaling pathway (**Figure 3D**). And the GSEA results suggested that myogenesis, epithelial-mesenchymal transition (EMT), apical junction, angiogenesis, and KRAS targets were the most significant enrichment items with the progression of tumor stages (**Figure 3E**).

**Figure 4:**
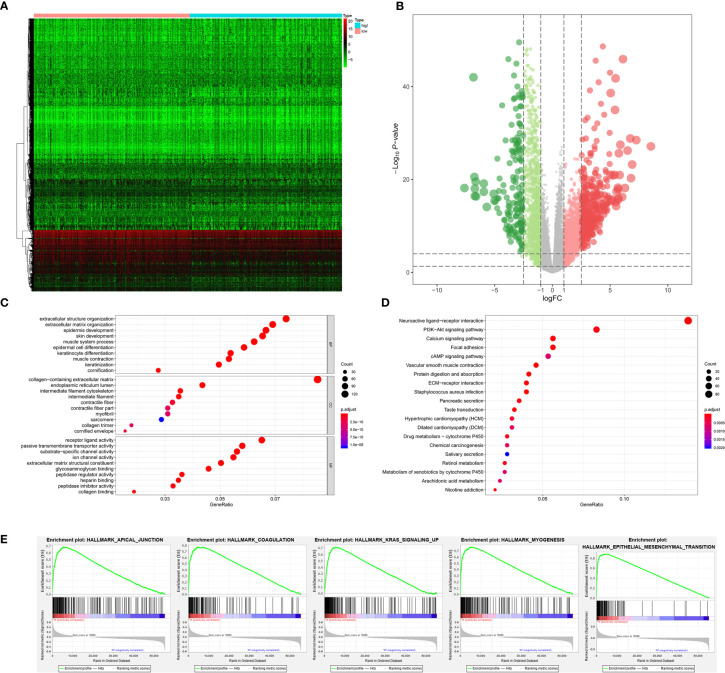
The results of differential expression genes analysis and functional enrichment analysis between low mRNAsi BLCAs and high mRNAsi BLCAs: The heatmap **(A)** and volcano plot **(B)** of the differential expressed genes. The GO **(C)**, KEGG **(D)**, and **(E)** top five GSEA enriched terms for the differential expressed genes.

The stemness DEGs was identified by low mRNAsi BLCAs and high mRNAsi BLCAs (divided by the median mRNAsi). Then, a total of 3,680 stemness DEGs including 1,403 downregulated ones and 2,277 upregulated ones were found (**Figures 4A, B**). Extracellular structure organization, collagen-containing extracellular matrix, receptor ligand activity were the most significant enrichment items of BPs, CCs, MFs associated with stemness (**Figure 4C**). Neuroactive ligand-receptor interaction, PI3K-Akt signaling pathway and calcium signaling pathway were stemness-associated KEGG pathways (**Figure 4D**). Similarly, the myogenesis, epithelial-mesenchymal transition (EMT), apical junction, and KRAS targets were also identified as most significant enrichment items related to mRNAsi in GSEA (**Figure 4E**).

**Figure 5:**
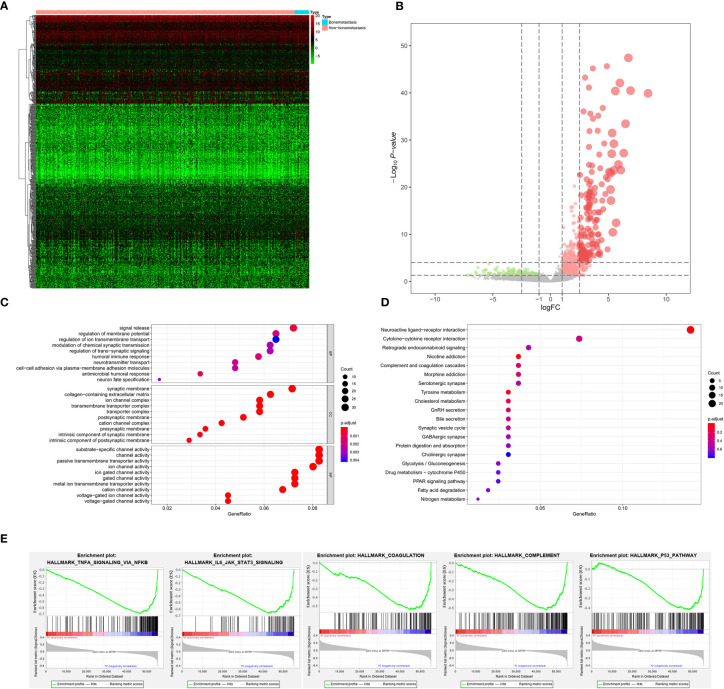
The results of differential expression genes analysis and functional enrichment analysis between BLCAs with and without bone metastasis: The heatmap **(A)** and volcano plot **(B)** of the differential expressed genes. The GO **(C)**, KEGG **(D)**, and **(E)** top five GSEA enriched terms for the differential expressed genes.

As for bone metastasis, the dataset consisted of 389 primary BLCAs without bone metastasis and 22 primary BLCAs with bone metastasis. The DEGs were also explored between them and we identified a total of 716 bone metastasis-associated DEGs including 143 downregulated genes and 573 upregulated genes. The heatmap and volcano plot were shown in **Figures 5A, B**. The bone metastasis-associated DEGs enriched in the GO items of signal release, synaptic membrane and substrate-specific channel activity (**Figure 5C**). KEGG items of neuroactive ligand-receptor interaction and cytokine-cytokine receptor interaction were also significantly enriched in the bone metastasis-specific DEGs (**Figure 5D**). And the enrichment plots of top five significant Hallmark gene set in GSEA were shown in **Figure 5E**, showing that TNFA targets, IL6-JAK-STAT3 signaling, coagulation, complement, and P53 targets were the most significant enrichment items related to bone metastasis of BLCA.

### The Clinic Correlation of mRNAsi

**Figure 6:**
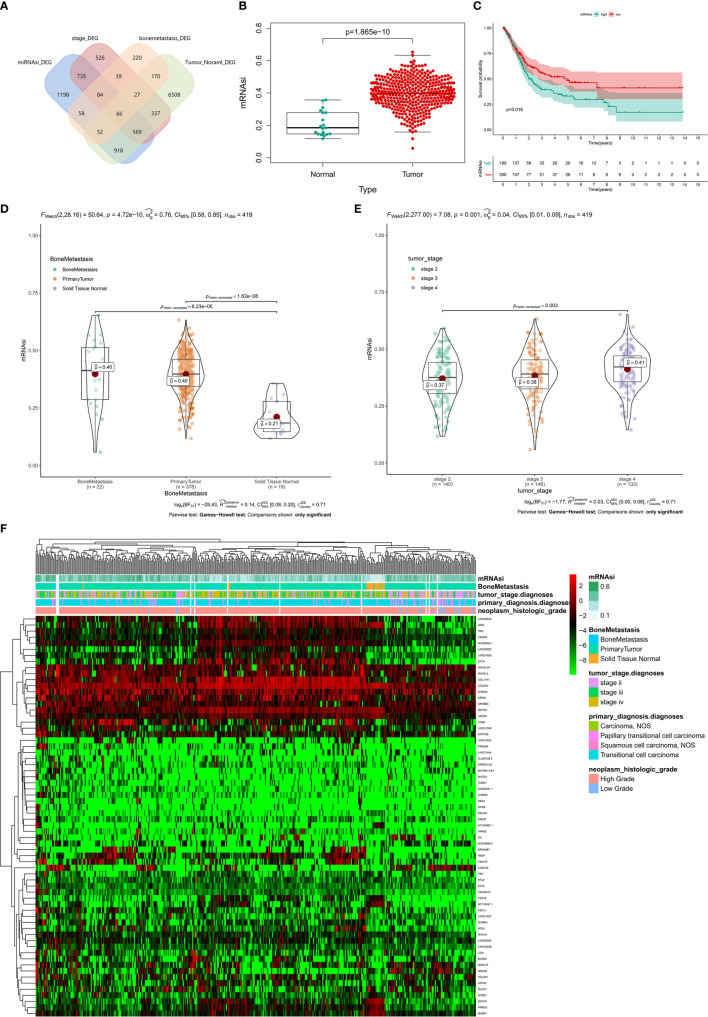
The clinical relevance of mRNAsi and identification of stemness-related genes. **(A)** The Venn plot of the tumorigenesis-, stemness-, stage- and bone metastasis-related differential expressed genes. **(B)** The difference of mRNAi between normal and tumor group. **(C)** Kaplan-Meier survival analysis of mRNAsi between normal and tumor group. **(D)** The difference of mRNAi among normal, tumor and bone metastasis. **(E)** The difference of mRNAi among different clinical stage. **(F)** The heatmap of mRNAsi, bone metastasis, tumor stage, primary diagnosis and neoplasm histologic grade.

Totally, 66 genes in the four groups of DEGs analysis were intersected by Venn plot and 13 genes were significantly up-regulated in the four DEG group (**Figure 6A**). The results of non-parametric tests (Mann-Whitney U-test or Kruskal-Wallis H-test) and Kaplan-Meier survival analysis suggested that compared with the normal solid tissue, mRNAsi was abnormally upregulated in the primary BLCA (P < 0.001, **Figure 6B**) and significantly associated with the prognosis of BLCA patients (P = 0.016, **Figure 6C**), bone metastasis diagnosis (P < 0.001, **Figure 6D**) and AJCC clinical stage (P < 0.001, **Figure 6E**). Besides, the expression levels of these 66 genes were presented in the heatmap (**Figure 6F**).

### The Identification of PRSGs and Independent Prognosis Analysis

**Figure 7:**
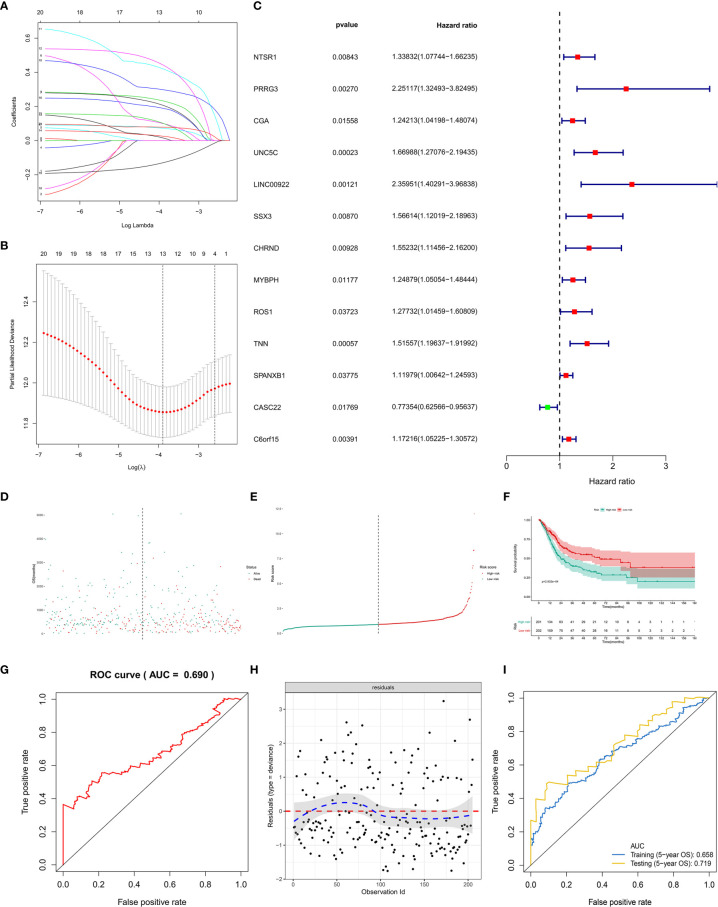
The model diagnosis of multivariate Cox model including prognostic stemness-related genes. **(A)** The final multivariate model of the 20 prognostic stemness-related genes. **(B–E)** The LASSO regression analysis of the model. **(F)** The Kaplan-Meier analysis of the risk score. The ROC curve **(G)** and residual plot **(H)** of the multivariate model. **(I)** And the five-fold cross-validation of the multivariate Cox model was performed, also showing similar discriminations to the original model (AUC of training dataset (5−year OS) = 0.658; AUC of testing dataset (5−year OS) = 0.719).

First of all, 66 DEGs intersected by Venn plot were incorporated into the univariate Cox regression analysis. Then, 20 genes with prognostic values in the univariate Cox regression analysis were defined as PRSGs integrated into the LASSO regression analysis (**Figures 7A, B**), suggesting that only 13 PRSGs (NTSR1, PRRG3, CGA, UNC5C, LINC00922, SSX3, CHRND, MYBPH, ROS1, TNN, SPANXB1, CASC22, and C6orf15) were essential for model fitting (**Figure 7C**).

The PI for each BLCA patient was calculated by the formula described in the methodology. The distribution of PI among all BLCA patients were shown by the risk line and risk scatterplot (**Figures 7D, E**). The Kaplan-Meier survival curve suggested that PI had prognostic value for BLCA patients (**Figure 7F**, P < 0.001). Moreover, the ROC of five-year of OS (AUC = 0.699, **Figure 7G**) and the residual plot (**Figure 7H**) illustrated an acceptable discrimination and GOF of the multivariate Cox regression model. And the five-fold cross-validation of the multivariate Cox model was performed, also showing similar discriminations to the original model (AUC of training dataset (5−year OS) = 0.658; AUC of testing dataset (5−year OS) = 0.719) (**Figure 7I**).

### Construction of the Prognostic Nomogram

**Figure 8:**
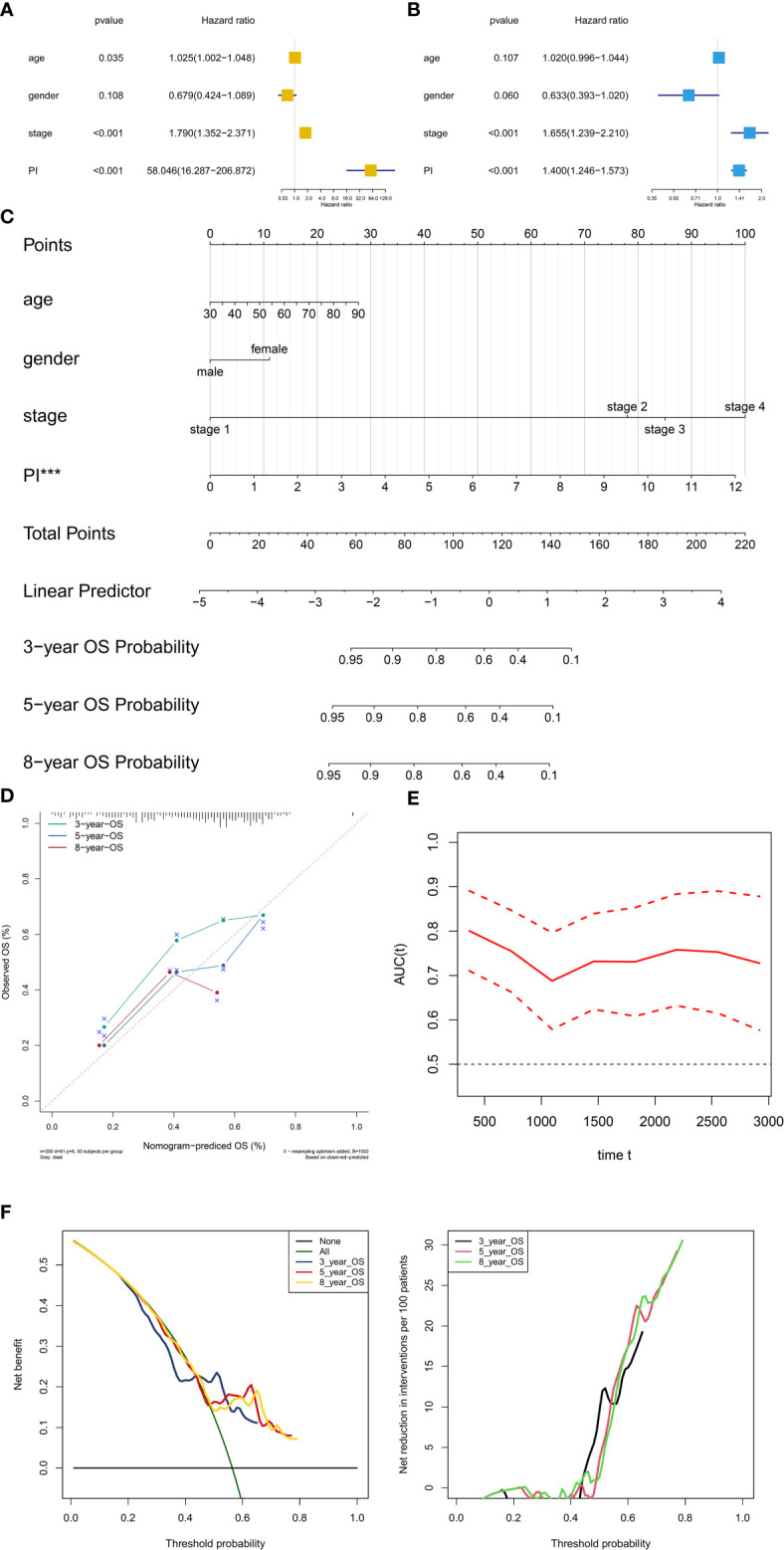
Independent prognosis analysis and construction of the prognostic nomogram. The univariate **(A)** and multivariate **(B)** Cox regression model corrected by demographics and stage. The constructed prognostic nomogram based on the multivariate Cox model **(C)**. The calibration curve illustrated acceptable calibration of the prognostic nomogram **(D)**. Additionally, the decision curve and time-related ROC with 95% confidence interval were also conducted to illustrate the patient benefit and the discrimination of the nomogram **(E, F)**. (***P < 0.001; **P < 0.01; **P < 0.05).

PI was then tested in the univariate and multivariate Cox model corrected by demographics and AJCC clinical stage. The results revealed that PI was an independent factor for predicting prognosis of BLCA in the univariate (HR = 60.735, 95%CI (17.376–212.289), P < 0.001, **Figure 8A**) and multivariate (HR = 1.412, 95%CI (1.256–1.558), P < 0.001, **Figure 8B**) Cox model. Besides, T stage and tumor grade were also integrated into univariate and multivariate Cox analyses to correct the PI, illustrating that PI was still an independent factor for predicting prognosis of BLCA in the univariate (HR = 1.456, 95%CI (1.315-1.612), P < 0.001, **Figure S1A**) and multivariate (HR = 1.480, 95%CI (1.324-1.654), P < 0.001, **Figure S1B**) Cox model.

Based on the Cox model, the prognostic nomogram was constructed to predict the 3-, 5-, and 8-year OS probability of BLCA patients (**Figure 8C**). The calibration curve illustrated acceptable calibration of the prognostic nomogram (**Figure 8D**). Additionally, the decision curve and time-related ROC with 95% confidence interval were also conducted to illustrate the patient benefit and the discrimination of the nomogram (When threshold probability of the nomogram was greater than 0.42, all patients could benefit from this model) (**Figures 8E, F**). Especially, as a clinical bioinformatics study, the gene expression levels should be corrected by demographics in multivariate regression models and age should to be treated as a categorical variable in the perspectives of some researchers. However, as age and gender were not significant in the multivariate Cox analysis, these two factors were inappropriate for nomogram construction. And to preserve more modeling samples, some other important clinicopathological features such as histology subtype, grade, pathologic T/N/M classification, and primary diagnosis were not included in the multivariate regression model. To further reduce these biases, the other five multivariate regression models were also constructed and diagnosed by calibration, time-related ROC and decision curve (**Figure S2**: modeling with 205 objectives same to **Figure 8** and treating age as a categorical variable) (**Figure S3**: modeling with all 403 objectives with all available variables and missing values) (**Figure S4**: modeling with all 403 objectives with all missing values and available variables except for age and gender) (**Figure S5**: modeling with 164 objectives with all available variables and excluding all missing values) (**Figure S6**: modeling with 164 objectives with all available variables except for age and gender and excluding all missing values). Especially, some paradoxical results could be found in these five multivariate regression models (e.g., malignant clinicopathological features achieved lower points in **Figure S3**; N1 achieved lower points than N0 in **Figure S3**). These problems might be caused by the several reasons. First, some missing values affected the fitting of the regression models, showing in the residual plots. Second, some clinicopathological features had endogenous interaction such as the AJCC stage was evaluated by T/N/M classification. Last but not least, although some variables contradicted to the clinical experiences in nomograms, these variables were not showing significant results in the multivariate regression models. Therefore, these variables could not be regarded as independent prognostic factors for BLCA patients, and were not suitable to illustrate in the nomograms. In order to distinguish the significant variables from the others, asterisks were used to annotate the statistical significance of each variables (***: P < 0.001; **: P < 0.01; **: P < 0.05). In sum, these five multivariate regression models and nomograms had limited clinical significance of predicting BLCA patients. However, PI was an independent factor in each multivariate regression model for predicting prognosis of BLCA.

### Identification of the PRSGs Co-Expressed Upstream TFs and the Downstream Signaling Pathways

**Figure 9:**
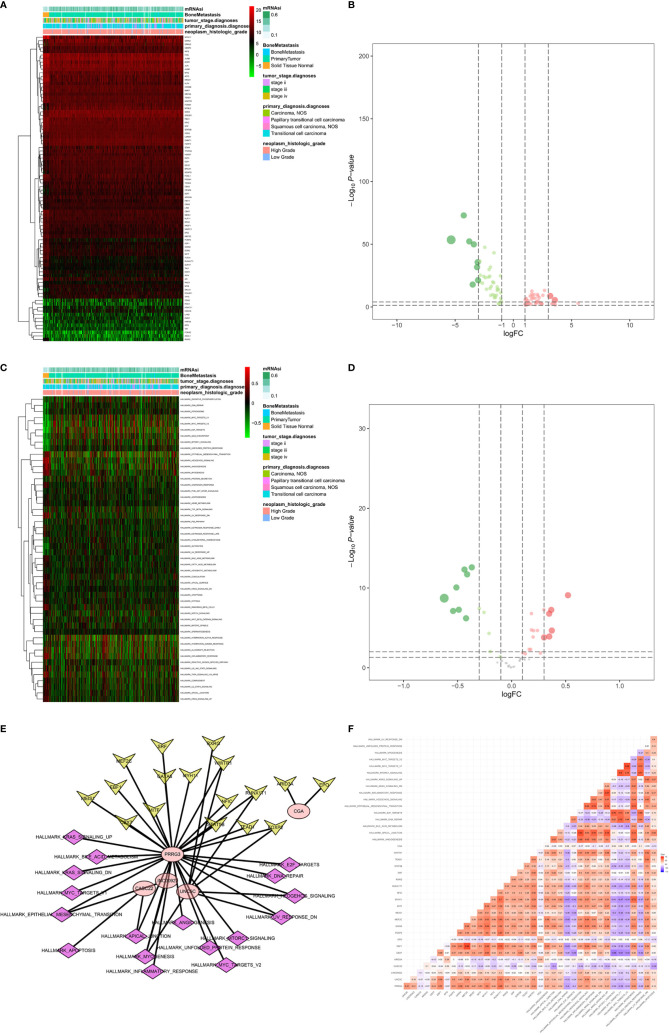
Identification of the PRSGs co-expressed upstream TFs, the downstream hallmarks of cancer gene sets. The heatmap **(A)** and volcano plot **(B)** of differentially expressed TFs between primary BLCAs and normal solid tissue samples. The heatmap **(C)** and volcano plot **(D)** of differentially expressed hallmarks of cancer gene sets between primary BLCAs and normal solid tissue samples. The point to point co-expression analysis **(E)** and co-expression interaction pairs **(F)** between TFs, PRSGs, and hallmarks of cancer.

A total of 86 differentially expressed TFs were identified between primary BLCAs and normal solid tissue samples. Their association with mRNAi, bone metastasis, tumor stage diagnoses, primary diagnosis, and neoplasm histologic grade were shown in the heatmaps (**Figure 9A**). The differentially expressed TFs were also presented in the volcano plots (**Figure 9B**). The relationships between 50 hallmarks of cancer gene sets and mRNAi, bone metastasis, tumor stage diagnoses, primary diagnosis, and neoplasm histologic grade were also shown in the heatmaps (**Figure 9C**). The volcano plots also described their expressions between primary BLCAs and normal solid tissue samples (**Figure 9D**). Among the above-mentioned 20 PRSGs, only four genes (CACNA1E, LINC01356, CGA, and SSX3) have consistent tendency in the four DEG group. Furthermore, point to point co-expression analysis were conducted among these four PRSGs, 86 TFs and absolute quantification of 50 hallmarks of cancer. Interaction pairs between TFs and PRSGs with |correlation coefficient| > 0.30 and P value < 0.05 along with interaction pairs between PRSGs and hallmarks of cancer with P value < 0.05 were used to construct the regulation network among TFs, four key PRSGs (CACNA1E, LINC01356, CGA, and SSX3) and hallmarks of cancer. In the heatmap of key hallmarks of cancer gene sets, we found the enrichment of oxidative phosphorylation, DNA repair, peroxisome, myc targets, E2F targets, mTORC1 signaling, unfolded protein response, cholesterol homeostasis, glycolysis, and UV response in the groups of tumorigenesis and bone metastasis. A total of 48 co-expression interaction pairs between TFs and PRSGs, and two co-expression interaction pairs between PRSGs and hallmarks of cancer passed the criteria and were used to construct the regulation network (**Figures 9E, F**). The regulation networks implied the correlation among CACNA1E, SSX3, LINC01356, DNA repair, myc targets, E2F targets, mTORC1 signaling, and unfolded protein response, which may regulate the tumorigenesis and bone metastasis of BLCA.

**Figure 10:**
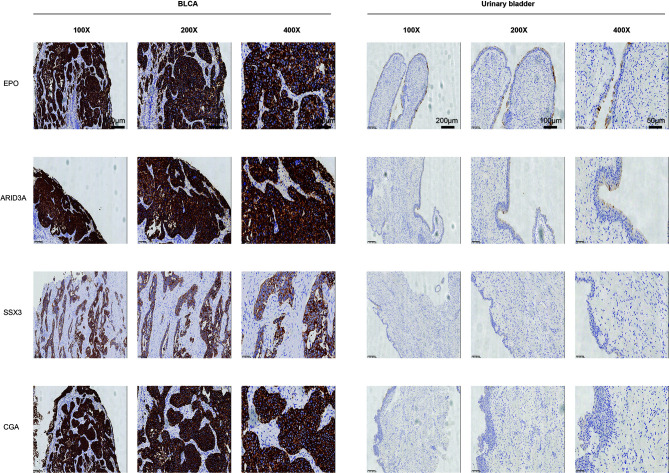
The protein levels of key TFs and PRSGs in BLCA and normal urinary bladder.

The protein levels of key TFs and PRSGs were further validated by IHC. The representative images of IHC revealed that the four proteins were highly expressed in BLCA by our samples (**Figure 10**). In the Human Protein Atlas, EPO was significantly higher in BLCA than that in normal urinary bladder (**Figure S7A**). The ARID3A and SSX3 highly expressed in BLCA, but barely found in normal urinary bladder (**Figure S7B, C**). The CGA was found in neither BLCA nor normal urinary bladder tissues (**Figure S7D**).

**Figure 11:**
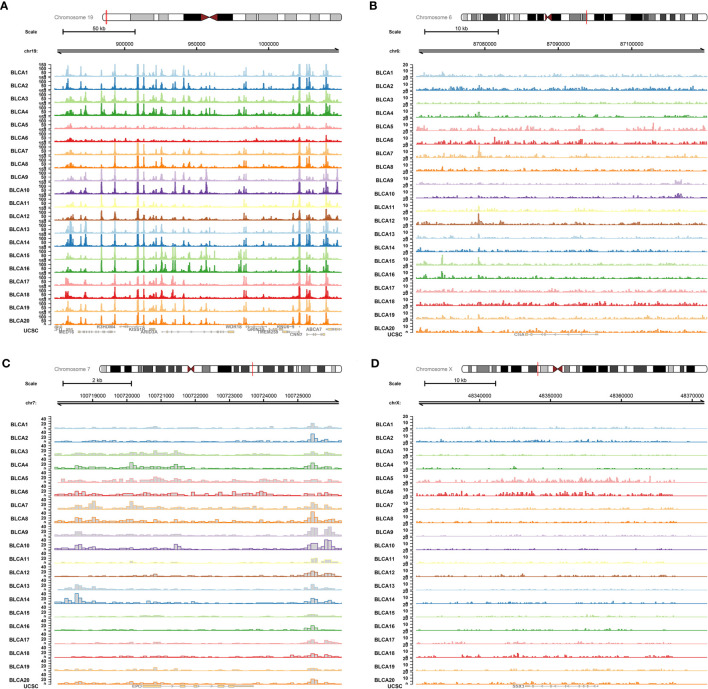
ATAC-seq validation. ATAC-seq data of BLCA were used to validated the regulation mechanism of four key PRSGs [ARID3A **(A)**, CGA **(B)**, EPO **(C)**, SSX3 **(D)**], illustrating their accessible peaks in the chromatin.

The ATAC-seq data of BLCA were further used to validate the regulation mechanism of four key PRSGs (ARID3A, **Figure 11A**; CGA, **Figure 11B**; EPO, **Figure 11C**; SSX3, **Figure 11D**), illustrating their accessible peaks in the chromatin.

## Discussion

Bladder cancer is a heterogeneous group of tumors with more than 40 histological subtypes of pathological patterns (2). BLCA is the common subtype, with a high metastasis rate (17). CSCs are considered responsible for many important aspects of tumors, such as tumorigenesis, progression and treatment recurrence, however, their roles in the tumorigenesis and metastasis have not been identified clearly in the BLCA. In this study, we identified mRNAsi as a reliable index for the tumorigenesis, prognosis, AJCC clinical stage and bone metastasis of BLCA. In addition, we found 20 key PRSGs which were associated with the tumorigenesis, clinical stage and bone metastasis. Based on them, a well-applied predict model was constructed which may assist urological surgeons in predicting the prognosis and bone metastasis of BLCA. Furthermore, based on multivariate Cox model and correlation analysis, CACNA1E, LINC01356, CGA, and SSX3 were inferred as potential diagnostic biomarkers and therapeutic targets for BLCA and its bone metastasis.

As a population of cancer cells, CSCs are regarded as the tumor initiating cells and therapeutic refractoriness cells (18). Nowadays, single-cell sequencing was used to identify the human bladder cancer stem cells (BCSCs) and uncovered 21 key altered genes in BCSCs (8). Many other studies also focused on the CSCs regulation in BLCA to investigate the potential mechanism and candidate targets (19–21). For example, CD24 could maintain the urothelial cancer stem-like traits and serve as a potential urinary biomarker for non-invasive BLCA (19). YAP was also regarded as a cancer stem cell regulator and a promising therapy target for patients with bladder cancer (20). Targeting COX2 and YAP1 pathways combined with systemic chemotherapy could also improve the clinical management of BLCA (21). However, the roles of CSCs in regulating the metastasis of BLCA, in especial bone metastasis, have not been described clearly.

In this study, we used the mRNAsi to identify the CSCs features in BLCA and found that it was significantly associated with the tumorigenesis, prognosis, AJCC clinical stage and bone metastasis in BLCA patients. It provided evidences for the roles of CSCs in the bone metastasis of BLCA, thus detecting and targeting key PRSGs may provide novel strategy for bone metastasis monitor and targeted therapy for patients with BLCA. In the multivariate Cox model and correlation analysis, we identified four key PRSGs, namely CACNA1E, LINC01356, CGA, and SSX3.

CACNA1E, the voltage-gated calcium channels (VGCCs) family member, often mediates the tumor evolution and heterogeneity formation *via* MAPK signaling pathway (22). Its amplification and overexpression were also found to be associated with the recurrence of Wilms’ tumors (23). CGA is the alpha-subunit of glycoprotein hormones. Its level was found to be elevated in many cancers and it could also participate in the process of tumor metastasis (24, 25). SSX3, one of the cancer/testis antigens, is also a well-known oncogene in many tumors. It was significantly associated with the poor outcome in patients with pancreatic ductal adenocarcinoma (PDAC) and could serve as a predictor of metastatic outcome in breast cancer patients (26, 27). Besides, SSX family can also serve as the vaccine targets for the treatment of sarcoma tumors (28).

Based on 20 PRSGs, we constructed a prediction model for the OS of patients with BLCA and the model achieved a good accuracy and applicability (AUC: 0.699). The construction of prediction model can assist oncologists in clinical decision-marking, thus many previous studies have focused on the identification of prognostic biomarkers in patients with BLCA (29–31). A lot of statistical methods, such as deep learning, Cox regression and LASSO regression analysis, have been used in the identification of the prognostic factors including the clinical information (age, clinical stage, and lymphovascular invasion), laboratory examination (C-reactive protein); molecular features (competing endogenous RNA, immune infiltration) (29, 30, 32–34). Although these results revealed the feasibility of personalized risk factors identification in predicting the prognosis of BLCA, none of them included the CSC-related signatures and PSRGs. Thus, the present research is a supplemental to the existing studies about the prognosis evaluation in patients with BLCA.

Due to the significant association between CSCs characteristics and TFs activity, epigenetic state, chromatin regulators and microenvironment cell networks (35), exploring the upstream TFs and downstream signaling pathways may offer more information of tumorigenesis and bone metastasis of BLCA. In this study, we identified the regulation networks between the abovementioned PRSGs and key TFs (EPO, ARID3A), hallmarks of cancer gene sets (DNA repair, myc targets, E2F targets, mTORC1 signaling, and unfolded protein response). EPO and its receptor have been reported to promote the tumor growth and invasion *via* an angiogenic effect (36). In tumor metastasis, EPO also plays an important role and may regulate the JAK/STAT and ERK1/2 pathways (37). ARID3A, a member of ARID family of DNA-binding proteins, serves as an independent predictor for prognosis in various cancers (38). It could control the tumor growth in a p53-dependent manner and promote esophageal squamous cell carcinoma invasion and metastasis (38, 39).

These five hallmarks of cancer gene sets generally took part in the tumor occurrence and development. DNA damage repair genes may be associated with the tumorigenesis and metastasis of prostate cancer (40). Besides, overexpression of c-MYC also leads to many cancers and it could be used as a possible target for therapeutic intervention in metastatic cancers (41). mTORC1, a well-known signaling pathway influenced by nutrients and growth factors, is significantly linked with metabolic diseases including cancer (5). Therapeutically, inhibition of mTOR signaling with rapamycin have been reported to attenuate the migration and invasion of colorectal cancer (42). Thus, we supposed that these identified signaling pathways may take part in the regulation of BLCA bone metastasis.

Although our data provide a reliable prediction model for BLCA and identify the potential mechanism for BLCA bone metastasis, this study still possessed some limitations that warrant consideration. Firstly, the samples involved in this study are from America, and thus the applicability of prediction model in European and Asian still needs further validation. Second, the sequencing data rely on one single cohort and the sample size is relatively limited. Third, the potential mechanism is based on bioinformation analysis and has not been verified by molecular and animal experiments. Thus, the future study will be conducted to verify these potential mechanisms *via* molecular experiments.

## Conclusion

Our study identifies mRNAsi as a reliable index in predicting the tumorigenesis, bone metastasis and prognosis of patients with BLCA and provides a well-applied model for predicting the OS for patients with BLCA. Besides, we also inferred the potential regulatory network between key PSRGs and cancer gene sets in mediating the BLCA bone metastasis.

## Data Availability Statement

The code and datasets generated and/or analyzed during the current study are available in the **Supplementary Material** and TCGA program (https://portal.gdc.cancer.gov).

## Author Contributions

YK, XZ, and XJW designed and performed the research, analyzed and interpreted the data, and drafted the manuscript. SL, MJ, LZ, XYW, and TZ collected the data and designed the methodology. JZ, JL, and DZ reviewed the manuscript. All authors contributed to the article and approved the submitted version.

## Funding

This study was supported by grants from the Zhejiang Provincial Health Bureau Science Foundation of China (NO. 2018KY017 and 2020KY408) and the Medical Health Science and Technology Project of Zhejiang Provincial Health Commission (NO. 2020ky040).

## Conflict of Interest

The authors declare that the research was conducted in the absence of any commercial or financial relationships that could be construed as a potential conflict of interest.

## Supplementary Material

Supplementary Figure 1 Independent prognosis analysis with T stage and tumor grade integrated into univariate and multivariate Cox analyses. T stage and tumor grade were also integrated into univariate and multivariate Cox analyses to correct the PI in the univariate (HR = 1.456, 95%CI (1.315-1.612), P < 0.001, **A**) and multivariate (HR = 1.480, 95%CI (1.324-1.654), P < 0.001, **B**) Cox model.

Supplementary Figure 2 Construction of the prognostic nomogram with 205 objectives and treating age as a categorical variable. **(A)** The constructed prognostic nomogram based on the multivariate Cox model. **(B)** The calibration curve illustrated acceptable calibration of the prognostic nomogram. The time-related ROC with 95% confidence interval **(C)**, decision curve **(D)** and residual plot **(E)** were also conducted to illustrate the patient benefit and the discrimination of the nomogram. Note: (***P < 0.001; **P < 0.01; **P < 0.05).

Supplementary Figure 3 Construction of the prognostic nomogram with all 403 objectives with all available variables and missing values. **(A)** The constructed prognostic nomogram based on the multivariate Cox model. **(B)** The calibration curve illustrated acceptable calibration of the prognostic nomogram. The time-related ROC with 95% confidence interval **(C)**, decision curve **(D)** and residual plot **(E)** were also conducted to illustrate the patient benefit and the discrimination of the nomogram. (***P < 0.001; **P < 0.01; **P < 0.05).

Supplementary Figure 4 Construction of the prognostic nomogram with all 403 objectives with all missing values and available variables except for age and gender. **(A)** The constructed prognostic nomogram based on the multivariate Cox model. **(B)** The calibration curve illustrated acceptable calibration of the prognostic nomogram. The time-related ROC with 95% confidence interval **(C)**, decision curve **(D)** and residual plot **(E)** were also conducted to illustrate the patient benefit and the discrimination of the nomogram. (***P < 0.001; **P < 0.01; **P < 0.05).

Supplementary Figure 5 Construction of the prognostic nomogram with 164 objectives with all available variables and excluding all missing values **(A)** The constructed prognostic nomogram based on the multivariate Cox model. **(B)** The calibration curve illustrated acceptable calibration of the prognostic nomogram. The time-related ROC with 95% confidence interval **(C)**, decision curve **(D)** and residual plot **(E)** were also conducted to illustrate the patient benefit and the discrimination of the nomogram. Note: (***P < 0.001; **P < 0.01; **P < 0.05).

Supplementary Figure 6 Construction of the prognostic nomogram with 164 objectives with all available variables except for age and gender and excluding all missing values **(A)** The constructed prognostic nomogram based on the multivariate Cox model. **(B)** The calibration curve illustrated acceptable calibration of the prognostic nomogram. The time-related ROC with 95% confidence interval **(C)**, decision curve **(D)** and residual plot **(E)** were also conducted to illustrate the patient benefit and the discrimination of the nomogram. (***P < 0.001; **P < 0.01; **P < 0.05).

Supplementary Figure 7 The protein levels of key TFs and PRSGs in BLCA and normal urinary bladder in the Human Protein Atlas. The representative IHC images of EPO **(A)**, ARID3A **(B)**, SSX3 **(C)** and CGA **(D)** in BLCA and normal urinary bladder tissues.
