# Supplementary figures and images for: Identification and Validation of the Prognostic Stemness Biomarkers in Bladder Cancer Bone Metastasis
Source: Front Oncol. 2021 Mar 19;11:641184. doi: 10.3389/fonc.2021.641184 (PMC8017322; doi:10.3389/fonc.2021.641184)

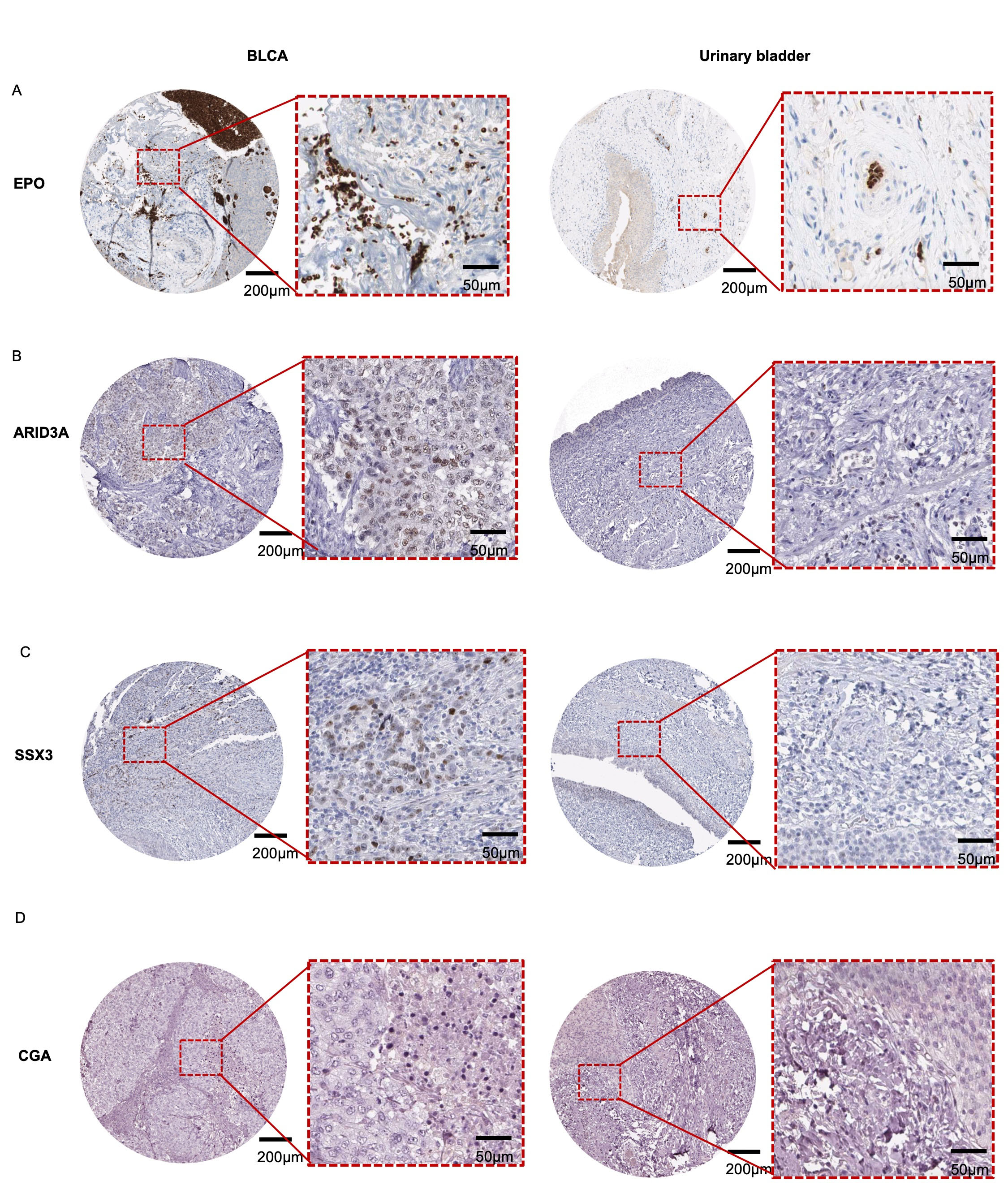

Supplement: Supplementary Figure 7 — The protein levels of key TFs and PRSGs in BLCA and normal urinary bladder in the Human Protein Atlas. The representative IHC images of EPO (A), ARID3A (B), SSX3 (C) and CGA (D) in BLCA and normal urinary bladder tissues. [file Image_7.jpeg]
